# *Pcolce2* overexpression promotes supporting cell reprogramming in the neonatal mouse cochlea

**DOI:** 10.1111/cpr.13633

**Published:** 2024-03-25

**Authors:** Changling Xu, Liyan Zhang, Yinyi Zhou, Haoliang Du, Jieyu Qi, Fangzhi Tan, Li Peng, Xingliang Gu, Nianci Li, Qiuhan Sun, Ziyu Zhang, Yicheng Lu, Xiaoyun Qian, Busheng Tong, Jiaqiang Sun, Renjie Chai, Yi Shi

**Affiliations:** ^1^ Health Management Center, Sichuan Provincial People's Hospital, School of Medicine University of Electronic Science and Technology of China Chengdu China; ^2^ Sichuan Provincial Key Laboratory for Human Disease Gene Study and Department of Laboratory Medicine, Sichuan Provincial People's Hospital, School of Medicine University of Electronic Science and Technology of China Chengdu China; ^3^ Research Unit for Blindness Prevention of the Chinese Academy of Medical Sciences (2019RU026) Sichuan Academy of Medical Sciences Chengdu Sichuan China; ^4^ State Key Laboratory of Digital Medical Engineering, Department of Otolaryngology Head and Neck Surgery, Zhongda Hospital, School of Life Sciences and Technology, School of Medicine, Advanced Institute for Life and Health, Jiangsu Province High‐Tech Key Laboratory for Bio‐Medical Research Southeast University Nanjing China; ^5^ Department of Otolaryngology‐Head and Neck Surgery The Affiliated Drum Tower Hospital of Nanjing University Medical School, Jiangsu Provincial Key Medical Discipline Laboratory Nanjing China; ^6^ Department of Neurology, Aerospace Center Hospital, School of Life Science Beijing Institute of Technology Beijing China; ^7^ Co‐Innovation Center of Neuroregeneration Nantong University Nantong China; ^8^ Otovia Therapeutics Inc Suzhou China; ^9^ Department of Otolaryngology, Head and Neck Surgery The First Affiliated Hospital of Anhui Medical University Hefei Anhui China; ^10^ Department of Otolaryngology‐Head and Neck Surgery, The First Affiliated Hospital of USTC, Division of Life Sciences and Medicine University of Science and Technology of China Hefei Anhui China; ^11^ Department of Otolaryngology Head and Neck Surgery, Sichuan Provincial People's Hospital University of Electronic Science and Technology of China Chengdu China; ^12^ Southeast University Shenzhen Research Institute Shenzhen China

## Abstract

Hair cell (HC) damage is a leading cause of sensorineural hearing loss, and in mammals supporting cells (SCs) are unable to divide and regenerate HCs after birth spontaneously. Procollagen C‐endopeptidase enhancer 2 (*Pcolce2*), which encodes a glycoprotein that acts as a functional procollagen C protease enhancer, was screened as a candidate regulator of SC plasticity in our previous study. In the current study, we used adeno‐associated virus (AAV)‐ie (a newly developed adeno‐associated virus that targets SCs) to overexpress *Pcolce2* in SCs. AAV‐*Pcolce2* facilitated SC re‐entry into the cell cycle both in cultured cochlear organoids and in the postnatal cochlea. In the neomycin‐damaged model, regenerated HCs were detected after overexpression of *Pcolce2*, and these were derived from SCs that had re‐entered the cell cycle. These findings reveal that *Pcolce2* may serve as a therapeutic target for the regeneration of HCs to treat hearing loss.

## Graphical abstract

Our study found that AAV‐*Pcolce2* promoted the re‐entry of SCs into the cell cycle in cultured cochlear organ tissues and in the postnatal cochlea, and regenerating HCs were detected in a neomycin injury model after overexpression of *Pcolce2*, which were derived from re‐entering cell cycle SCs.

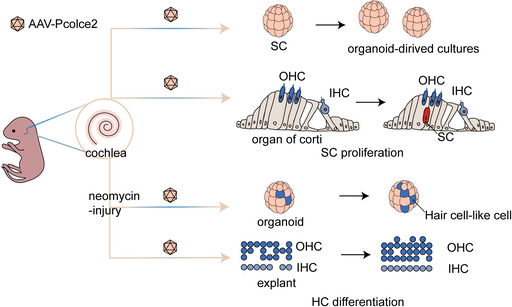

## 1 INTRODUCTION

More than 150 million people worldwide suffer from hearing loss. According to the latest World Health Organization report, hearing loss is a major global health problem, currently the third leading cause of disability and first among sensory impairments.^1^ While damage and death of sensory hair cells (HCs) and spiral ganglion neurons are the main causes of hearing loss in sensorineural hearing loss (SNLH).^2^, ^3^ Although cochlear implants and hearing aids are currently the main clinical treatment for severe hearing loss, with a deeper understanding of the molecular basis of auditory development, much research has been devoted to HC regeneration and neuronal repair in the hope of restoring sensation to the inner ear at a fundamental level.^4^

In vertebrates other than mammals, such as birds and fish, HCs can be regenerated from supporting cells (SCs), but in mammals, HCs cannot spontaneously regenerate in the mature cochlea.^5^ Recent studies have shown, however, that mammalian HCs can be induced to regenerate under certain conditions; for example, Lgr5‐labelled SCs can respond to Wnt signals and proliferate and differentiate into HCs.^6^, ^7^ HCs and SCs have a close lineage relationship during the development of the auditory epithelium,^8^, ^9^ and SCs can act as inner ear progenitor cells for self‐renewal in vitro.^10^ That SCs can divide or transdifferentiate into HCs has been demonstrated in several studies,^7^, ^11^, ^12^, ^13^ and this process involves several signalling pathways. For example, *Atoh1* overexpression promotes the generation of ectopic HCs,^14^, ^15^ it also stimulates the regeneration of HCs from Sox2^+^ SCs by promoting the Wnt/beta‐catenin pathway,^16^, ^17^ and enhances the regeneration of HCs from SCs when coupled with inhibition of the Notch pathway.^18^, ^19^ Research has also indicated that upregulation of the Hedgehog pathway promotes inner ear progenitor cell proliferation and HC formation in mice.^20^, ^21^ However, considering that the maturity of the regenerated HCs is still far from that of native HCs, it is necessary to identify new genes for HC regeneration.

Lgr5^+^ progenitors are considered to be inner ear stem cells and have great potential to proliferate and differentiate into HCs.^7^, ^22^ In early studies, our team analysed the differences in Lgr5^+^ progenitors in neomycin‐injured and non‐injured animals^23^, ^24^ and then screened for factors that regulate HC regeneration. Our results revealed that a novel gene, *Pcolce2* (procollagen C‐endopeptidase enhancer 2), could promote the proliferation of inner ear stem cells.^23^, ^24^ *Pcolce2* is a collagen‐binding protein that acts as a functional procollagen C protease enhancer and also has heparin‐binding activity.^25^ *Pcolce2* is also associated with high‐density lipoprotein uptake^26^, ^27^, ^28^ and is required for efficient procollagen processing and fibrillar collagen deposition in chronically pressure‐overloaded myocardium.^29^ Studies have also shown that *Pcolce2* can stimulate reactive oxygen species production in neutrophils.^30^ In this study, we found that the expression of PCOLCE2 in the mice's inner ear initially increased after birth and then gradually decreased with age, and we hypothesised that *Pcolce2* is involved in the development of HCs and SCs in the cochlea of mice.

Gene therapy for hearing disorders is the subject of many research efforts, and AAV (adeno‐associated virus) is widely used for gene therapy due to its low toxicity, high infection rate, sustained targeted gene expression, rapid and easy production, etc.^31^, ^32^, ^33^, ^34^ Previous studies have shown that AAV‐ie can safely and effectively transfect inner ear SCs,^35^ thus it can be used as a gene delivery vector for inner ear progenitor cells. In this study, we used AAV‐ie as a delivery vehicle and constructed AAV‐ie‐*Pcolce2* (AAV‐*Pcolce2*) to overexpress *Pcolce2* in inner ear SCs. We found that *Pcolce2* overexpression promoted cochlear organoid formation in two‐dimensional (2D) and three‐dimensional (3D) culture environments, and SC proliferation was also observed after *Pcolce2* overexpression in neonatal mice. After aminoglycoside exposure, overexpression of *Pcolce2* in combination with Wnt activation and inhibition of Notch signalling led to significant HC differentiation. Thus, our results suggest a potential role for *Pcolce2* in the regulation of HC regeneration.

## 2 MATERIALS AND METHODS

### 2.1 Animals

FVB mice were used as wild‐type (WT) mice in the experiments. Sox9^CreER^ and Rosa26‐tdTomato^loxp/loxp^ mice were acquired from Jackson Laboratory. Sox9^CreER^/Rosa26‐tdTomato^loxp/loxp^ mice were derived from the cross between Sox9^CreER^ and Rosa26‐tdTomato^loxp/loxp^ mice. Experiments used both male and female mice. On the first day after birth (P1), the mice were intraperitoneally injected with tamoxifen (Sigma, 0.75 mg/10 g body weight in corn oil) to activate Cre recombinase. AAVs were then injected using a round window membrane injection (RWM), and cochleae were harvested on different days after the AAV injection. Protocols approved by the Southeast University Animal Care and Use Committee were used for all animal procedures. Genotyping primers are shown in Table S1.

### 2.2 2D stem cell culture experiment

For the 2D culture, AAVs were first injected into P1 mice via the RWM. The dissociated P3 cochlear basement membrane was placed in hanks' balanced salt solution and digested into single‐cell suspension with 0.25% trypsin–ethylene diamine tetraacetic acid (EDTA) (Thermo Fisher) and 1% DNase I (Sigma). An equal volume of 5 mg/mL trypsin inhibitor (Worthington Biochem) was added to terminate the digestion. Then, the single‐cell suspension was prepared by filtering through a cell filter of 40 μm in diameter (Falcon), centrifuging and resuspending in a 2D medium consisting of dulbecco's modified eagle's medium (DMEM)/F12 (Thermo Fisher), 2% B27 (Stem Cell), 1% N2 (Invitrogen), 20 ng/mL epidermal growth factor (EGF; Stem Cell), 50 ng/mL insulin‐like growth factor (IGF; Sigma), 0.1% ampicillin (Sangon Biotech) and 10 ng/mL β‐fibroblast growth factor (β‐FGF; Stem Cell). The cells were seeded in 96‐well transparent flat‐bottomed microplates with ultra‐low attachment force (Corning) at 5000 cells/well and then cultured for 5 days, with fresh medium added daily. Five days later, images were captured on the live cell workstation (Zeiss) and the cells were fixed with 4% PFA for subsequent immunofluorescence staining.

### 2.3 3D inner ear organoid culture

For the 3D culture, the cochlear basement membrane was collected from P1 to P2 mice, and the digestion procedure for the basement membrane was the same as for the 2D culture. Cells were resuspended in a 1:3 mixture of Matrigel gel matrix (Corning) and 3D proliferation medium. Approximately, 5000 cells were plated on a 9 mm round slide in a 24‐well plate. After solidifying at 37°C, a 3D proliferation medium was added for culture. The total amount of virus was 2 × 10^10^ genomic copies (GCs) per well. The components of the proliferation medium were DMEM/F12 medium, 2% B27, 1% N2, 20 ng/mL EGF, 50 μg/mL IGF, 10 g/mL β‐FGF, 3 μM CHIR99021 (Sigma), 1 mM VPA (Sigma), 2 μM 616452 (Sigma) and 1% ampicillin. Before collection, samples were incubated with 3 μM 5‐ethynyl‐2‐deoxyuridine (EdU) (Thermo Fisher) for 1 h. After 7–8 days of proliferation culture, the medium was replaced with a differentiation medium consisting of 1% N2, 2% B27, 3 μM CHIR99021, 5 μM LY411575 (Sigma), 1% ampicillin and DMEM/F12 for 10 days.

### 2.4 Inner ear explant culture and injury model construction

The cochlear basement membrane was dissected from P1 to P2 mice, and to improve the transfection efficiency, it was evenly cut into two segments with a scalpel, and then attached to a slide before coating them with Cell‐Tak (Corning) for culture. Neomycin (1 mM, Sigma) was used to injure HCs, and AAVs were added simultaneously for 12 h, and then the EdU was added for 3 days. The composition of the basal medium was DMEM/F12, 2% B27, 1% N2 and 0.1% ampicillin. Samples were collected after a total of 4 days in culture.

### 2.5 Virus packaging and purification

AAV‐ie viruses were packaged according to a previously published method,^35^ and co‐transfected HEK‐293T cells with a three‐plasmid system, including AAV‐ie, helper plasmid and the control target gene plasmid (CAG‐mNeonGreen‐NLS) or the experimental *Pcolce2* target gene plasmid (CAG‐Pcolce2‐mNeonGreen‐NLS). PEG8000 (Sigma) was used to concentrate and purify the cells. Viral titers were determined by quantitative real‐time polymerase chain reacations (qPCR) using SYBR QPCR Mix (Vazyme). The inverted terminal repeat sequences used for the primers are listed in Table S1.

### 2.6 RWM injection of AAVs

Newborn mice were anaesthetised on ice for 1–2 min and placed on ice for surgery. After anaesthesia, a small incision was made behind the left ear to expose the RWM. The injection was performed using glass pipettes (Drummond) with 25 mm tips driven by ultra‐micro‐pumps UMP3 (World Precision Instruments). The total AAV injection was approximately 1.5 μL. After the virus injection, veterinary tissue adhesive (Millpledge Veterinary) was used to glue the surgical wound. The surgical procedure was controlled to be completed within 5 min, and the pups were allowed to recover for another 10 min at 37°C before being returned to their mothers to continue nursing.

AAV was injected together with LY411575 and CHIR99021, using 30% PEG400 used for solubilisation, and the final concentration of AAV was 3 × 10^13^ GCs/mL, and the final concentrations of LY411575 and CHIR99021 were 5 and 4 mM.

### 2.7 Construction of the HC injury model in mice

After AAV injection via the RWM as described above, neomycin was injected subcutaneously in the back of the neck every day at 0.15 mg/g body weight daily from P8 to P14. Cochlear samples were collected on P21.

### 2.8 Immunofluorescence staining

Cochlear samples were fixed with 4% PFA followed by decalcification with 0.5 M EDTA (Biosharp). Cochlear samples were divided into three turns (apical, middle and basal) and attached to 9 mm round glass slides (Biosharp) using Cell‐Tak. Block the samples with 10% donkey serum, followed by primary antibodies such as Sox2 (R&D Systems, 1:400) and Myosin 7a (Proteus Bioscience, 1:1000) were incubated overnight. The next day, after washing with 0.5% PBS‐Triton X‐100 (Life Science), incubation with secondary antibodies was performed, including donkey anti‐rabbit 647 conjugate (Life Science), donkey anti‐mouse 647 conjugate (Life Science) and donkey anti‐goat 555 conjugate (Life Science). DAPI (Solarbio, 1:1000 dilution) was used to stain the nuclei. The samples were then washed with 0.5% PBS‐Triton X‐100 solution and mounted in a dako anti‐fluorescence quenching medium. EdU was stained using the EdU Imaging Kit (Life Science). All immunofluorescence images were obtained on an LSM 900 (Zeiss).

### 2.9 Quantitative real‐time PCR

RNA was extracted from the mouse cochlea and cells using Trizol reagent (Life Science), chloroform and isopropyl alcohol were used for lysis and purification, and a Nanodrop (Thermo Fisher) was used for concentration determination. After uniform calibration of the amount of RNA, reverse transcription was performed using a cDNA Synthesis Kit (Thermo Fisher). qPCR was performed using SYBR Master Mix (Vazyme) on a CFX96 (Bio‐Rad). Relative quantification was then calculated.

### 2.10 Western blot

Cochlear samples were lysed in protease‐inhibited RIPA buffer (Roche). The extracted protein was diluted in 5× sodium dodecyl sulfate‐polyacrylamide gel electrophoresis buffer (Beyotime), and the proteins were separated on 10% PAGE gels (Beyotime) and blotted onto a polyvinylidene difluoride membrane (Millipore). The samples were then incubated in 5% skim milk for 1–2 h followed by primary anti‐PCOLCE2 antibody (Cusabio, 1:500 dilution) overnight, and the internal reference was GAPDH (Thermo Fisher, 1:3000 dilution). After three washes with 1% phosphate buffered saline with tween, samples were incubated for 1–2 h with appropriate secondary antibodies, including horseradish peroxidase goat anti‐mouse IgG and anti‐rabbit IgG (H+L) (ABclonal). SuperSignal West Pico PLUS (Thermo Fisher) was used to expose the images on a Chemi Capture (Tanon, 5200), and the images were subjected to grayscale analysis in ImageJ.

### 2.11 Single‐cell data processing in the mouse cochlea

We downloaded single‐cell normalised expression profile datasets for time points P1 and P7 from the gene expression omnibus (GEO) database, with the accession number GSE137299. Using the Seurat software (Version 4.1.3, https://github.com/satijalab/seurat), we performed cell integration, normalisation, dimensionality reduction, clustering and cell type identification. First, we created a Seurat object using the ‘CreateSeuratObject’ function to import the downloaded cell‐by‐gene count expression matrices. The data were then normalised using Seurat's ‘ScaleData’ function. To integrate different expression datasets from the same time point and remove batch effects, we used Seurat's ‘FindIntegretedAnchors’ and ‘IntegretedData’ functions.

Linear dimensionality reduction was performed using Seurat's ‘RunPCA’ function, followed by non‐linear dimensionality reduction analysis using ‘RunUMAP,’ and then visualizing the cells on a 2D UMAP plot. Clustering was done by employing different ‘resolution’ values for P1 and P7 in Seurat's ‘FindClusters’ function (P1 = 0.5, P7 = 1.6). Cell markers for P1 and P7 time points^36^ were used to identify SC and HC cells. Finally, the expression levels of the *Pcolce2* gene in SC and HC at both time points were presented using Seurat's ‘VlnPlot’ function.

### 2.12 RNA sequencing

RNA was extracted from organoids using Trizol and analysed by NovoGene Inc. All mRNAs were analysed on an Illumina sequencing platform, and mapping of the differential genes was performed using R (version 3.0.3). Correlation analysis was based on the Pearson correlation between samples calculated from the FPKM expression matrix. Genes with |logFC| >1 were used to construct Venn diagrams. The significance of gene expression differences was analysed using DESeq2, and differential gene expression was visualised using volcano plots drawn with ggplot2. To screen for differentially expressed genes between the high and low expression groups, the cutoff values were |logFC| >1 and *p*adj <0.05.

### 2.13 Auditory brainstem response

Mice were injected intraperitoneally with sodium pentobarbital (10 mg/mL) at a dose of 1 mg/10 g body weight to induce deep anaesthesia. Closed‐field auditory brainstem response (ABR) thresholds were then measured in AAV‐injected mice using a TDT System III workstation (Tucker‐Davis Technologies). Electrodes were connected so that the positive electrode was on the top of the head, the ground wire was connected to the thigh and the negative electrode was under the ear. ABR testing was performed using 4, 8, 12, 16, 24 and 32 kHz tone tests in which the tone intensity was gradually reduced from 90 dB in 5 dB increments.

### 2.14 Statistical analysis

Immunofluorescence image processing was performed using the ImageJ software. Statistical methods used were two‐tailed Student's *t*‐test or one‐way analysis of variance with Tukey's multiple comparison test in GraphPad 7. All statistical results are presented as mean ± standard error of the mean, and all results with a *p‐value* above 0.05 were considered statistically significant.

## 3 RESULTS

### 3.1 AAV‐*Pcolce2* increases the proliferative capacity of HC progenitors in 2D and 3D organoids

To investigate the cochlear expression pattern of *Pcolce2*, western blot and immunofluorescence were used to demonstrate PCOLCE2 expression in mice cochlea of different ages (Figure S1). We found that PCOLCE2 was mainly expressed in HCs and was not observed in SCs (Figure S1A). To further validate the distribution, we downloaded the single‐cell normalised expression profiling dataset of mouse cochlea at time points P1 and P7 from the GEO database.^36^ Then, we performed cell integration, normalisation, dimensionality reduction, clustering and cell type identification. The expression of *Pcolce2* in the mouse cochlea was examined in HC and SC, confirming that it is predominantly expressed in HCs (Figure S1B). At the same time, western blot results showed that PCOLCE2 was expressed in different age groups and gradually decreased after P7 (Figure S1C,D).

**FIGURE 1:**
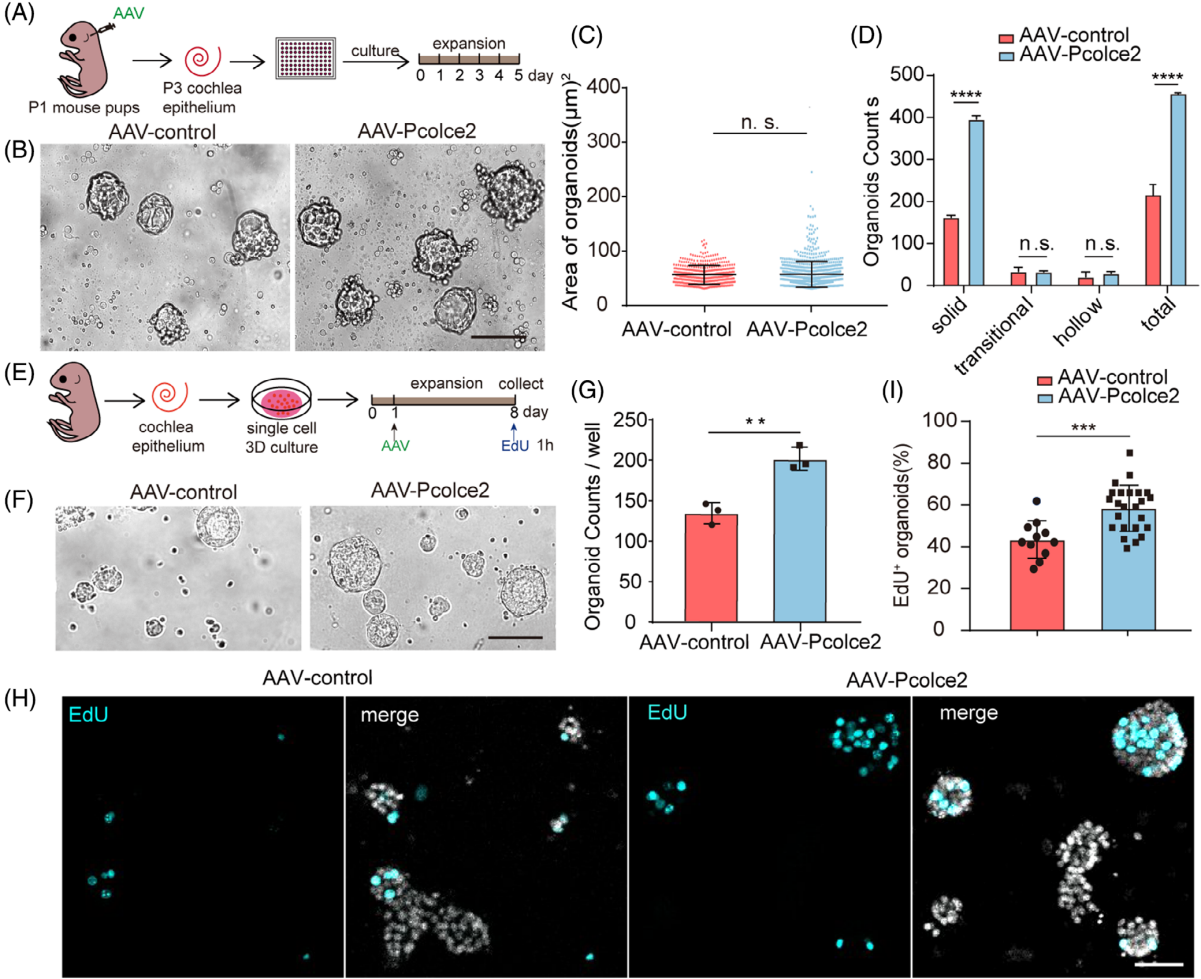
AAV‐procollagen C‐endopeptidase enhancer 2 (*Pcolce2*) promotes the expansion of cochlear progenitor cells in two‐dimensional (2D) and three‐dimensional (3D) cultures. (A) Experimental flow chart for the 2D culture of organoid. AAV‐control and AAV‐*Pcolce2* were injected into mice at P1, the basement membrane was dispersed into single cells at P3, and samples were cultured in vitro for 5 days. The dose of AAV: 4.5 × 10^10^ GCs/cochlea. (B) The organoids' brightfield (BF) images of AAV‐control and *Pcolce2*. Scale bar: 100 μm. (C, D) The statistics of organoids generated in (B) are shown in area (C) and number (D). (E) Experimental plan of the 3D cochlear organoid culture. The inner ear basement membranes of P1 mice were digested into single cells. AAVs were added on the first day at a dose of 2 × 10^10^ GCs/well, followed by the collection of samples and incubation with EdU for 1 h. (F) The organoids' brightfield images transformed by AAV‐control and *Pcolce2* in Matrigel. Scale bar: 100 μm. (G) The organoid counts per well in (F). (H) The organoids immunofluorescence images of AAV‐control and *Pcolce2*. EdU (cyan) marks proliferating cells, and DAPI (grey): nuclei. Scale bar: 200 μm. (I) The percentage of EdU^+^ organoids in (H). **p < 0.01, ***p < 0.001 and ****p < 0.0001, n.s. means no significance.

To explore the effect of overexpressing *Pcolce2* on HC progenitors, we used the AAV‐ie vector to transfect organoids. There was no statistical difference in the area of the organoid spheres in the HC progenitors in 2D suspension culture between AAV‐control and AAV‐*Pcolce2* (Figure 1A–C). Previous studies showed that solid‐type organoids have greater plasticity,^37^ and compared to AAV‐control, AAV‐*Pcolce2* induced more solid‐type organoids (Figure 1D), indicating that AAV‐*Pcolce2* can promote the proliferation of inner ear progenitors under 2D culture conditions. We further validated the effect of AAV‐*Pcolce2* overexpression under 3D culture conditions in Matrigel (Figure 1E). The number of organoids in each well of the AAV‐*Pcolce2* group increased significantly in comparison to the AAV‐control (Figure 1F,G). In addition, the proportion of EdU^+^ organoids increased significantly after *Pcolce2* overexpression (Figure 1H,I). These data suggest that *Pcolce2* can promote the proliferation of inner ear progenitors.

### 3.2 AAV‐*Pcolce2* enhances SC proliferation in vivo

**FIGURE 2:**
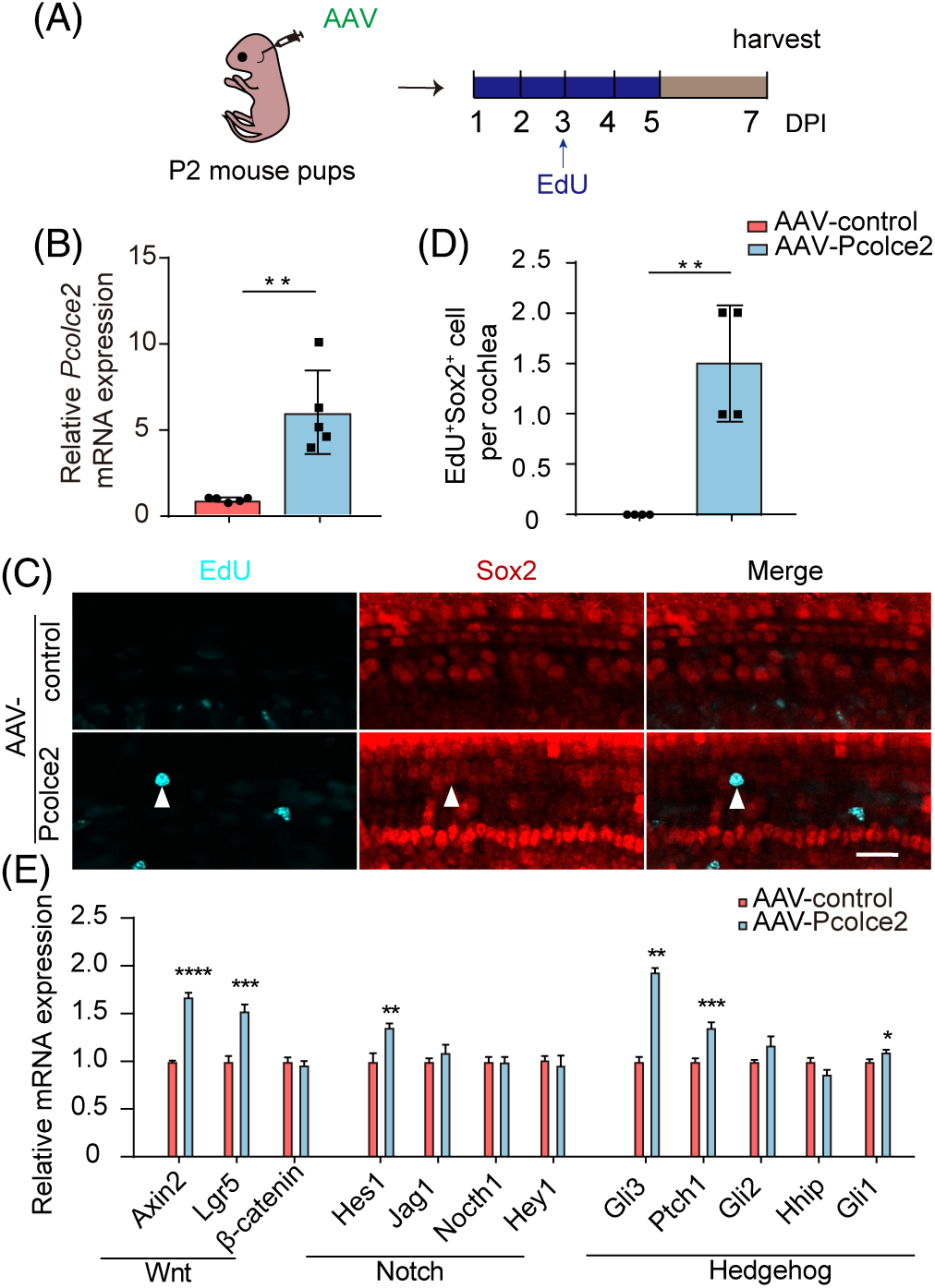
Procollagen C‐endopeptidase enhancer 2 (*Pcolce2*) promotes cochlear progenitor cell proliferation in vivo. (A) Experimental design. AAVs were injected in P2 mice, and EdU was injected on Days 1–5. DPI, days post‐injection. (B) The mRNA expression of the *Pcolce2* gene in the cochlea after virus injection. (C) The EdU (cyan) immunostaining images of AAV‐treated cochlear epithelia. White arrows indicate the EdU^+^/Sox2^+^ cells. The AAV dose was 9 × 10^10^ GCs/cochlea. Sox2 (red) marks the supporting cells, and AAV‐transfected cells are green. Scale bar: 50 μm. (D) EdU^+^/Sox2^+^ cell counts in (C). (E) The mRNA levels of different pathway‐related genes in AAV‐control and *Pcolce2*‐injected cochlea in (A). *p < 0.05, **p < 0.01, ***p < 0.001 and ****p < 0.0001.

To verify that the effects of *Pcolce2* were consistent in vivo and in vitro, AAVs were injected through the RWM in P2 mice (Figure 2A). The qPCR results verified the overexpression of *Pcolce2* in vivo (Figure 2B), and the EdU and Sox2 immunofluorescence staining results showed that *Pcolce2* overexpression induced more EdU^+^/Sox2^+^ cells in the cochlea compared with the control group (Figure 2C,D), suggesting a role for *Pcolce2* in promoting SC proliferation in vivo. We then detected the downstream genes related to the Wnt, Notch and Hedgehog signalling pathways and found that the expression of the Wnt target genes such as *Axin2* and *Lgr5*, the Notch signalling pathway‐related gene *Hes1* and the Hedgehog signalling‐related genes *Gli3* and *Ptch1* all increased compared with control (Figure 2E). Together, these data showed that the mechanism through which overexpression of *Pcolce2* induces SC proliferation might include the activation of the Wnt, Notch and Hedgehog pathways.

### 3.3 AAV‐*Pcolce2* does not promote HC differentiation under normal culture conditions

**FIGURE 3:**
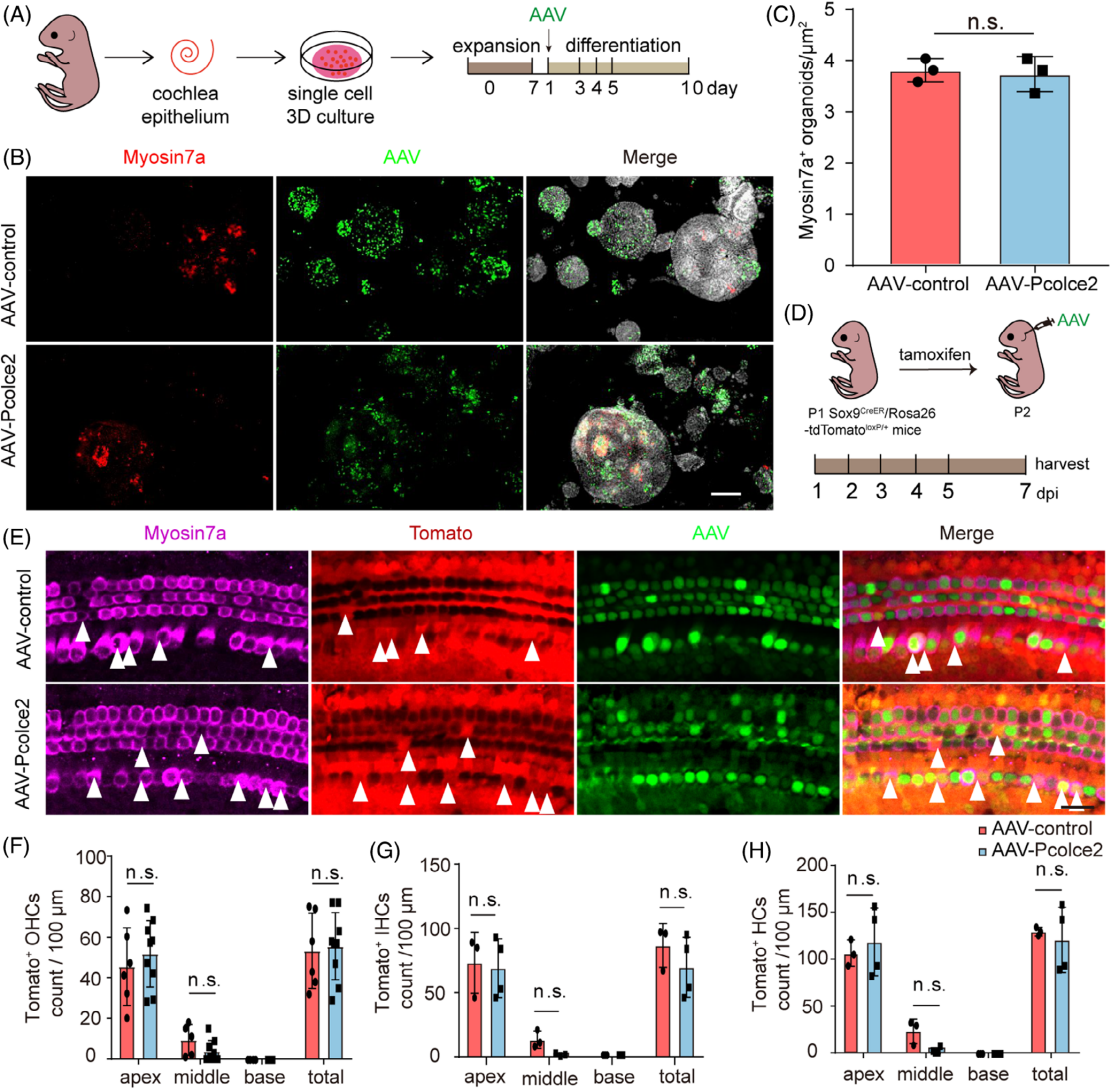
Procollagen C‐endopeptidase enhancer 2 (*Pcocle2*) does not affect the differentiation of inner ear progenitor cells. (A) Experimental plan. After 7 days of proliferation, AAV was added along with the differentiation medium, and the differentiation culture lasted 10 days. The AAV dose: 3 × 10^10^ GCs per well. (B) Immunostaining images of control and *Pcolce2*‐overexpressing organoids. AAV marks transduced cells (green), Myosin7a marks hair cell (HCs) (red), and DAPI labels nuclei (grey). Scale bar: 200 μm. (C) Myosin7a^+^ cells of organoids in (B). (D) Lineage tracing of Sox9^+^ supporting cells (SCs) in AAV‐control and *Pcolce2* cochlea. Tamoxifen was injected at P1, and 12 h later injected the AAV. (E) Representative images of Sox9^+^ SCs tracking by Tomato fluorescence. Myosin7a (shown in magenta) marks HCs. Myosin7a+/Tomato+ cells are indicated by white arrows. AAV dose: 9 × 10^10^ GCs/cochlea. Scale bar: 50 μm. (F–H) The number of Tomato+ outer HCs (OHCs) (F), Tomato+ inner HCs (IHCs) (G), and total tdTomato+ HCs (H) per cochlea. n.s. refers to no significance.

Next, we explored the role of *Pcolce2* in organoids under differentiation conditions in vitro (Figure 3A). Myosin7a staining showed no significant difference in the number of Myosin7a^+^ organoids in the AAV‐Control and AAV‐*Pcolce2* groups (Figure 3B,C), suggesting that the overexpression of *Pcolce2* has no obvious effect on the differentiation of inner ear stem cells.

We then tested the differentiation ability of *Pcolce2* overexpression in the mouse cochlea in vivo. After injecting the virus via the RWM into P2 neonatal mice, immunofluorescent staining for Myosin7a showed a few ectopic HCs in the cochlea of *Pcolce2* overexpressing mice (Figure S2). Sox9 is a marker of SCs in the cochlea and also labels inner ear stem cells,^38^, ^39^ and because stem cells of the inner ear are the HCs source and SCs, Sox9^CreER^/Rosa26‐tdTomato^loxp/loxp^ mice can be used to perform lineage tracing experiments in vivo (Figure 3D). Myosin7a^+^/Tomato^+^ cells were detected in the *Pcolce2* overexpressing cochlea (Figure 3E), but the numbers were not significantly different compared to control (Figure 3F–H). Together, these data show that *Pcolce2* can promote the proliferation of SCs in vivo and in vitro but has no obvious effect on the differentiation of SCs into HCs.

### 3.4 AAV‐*Pcolce2* promotes HC regeneration after cochlear basilar membrane damage

**FIGURE 4:**
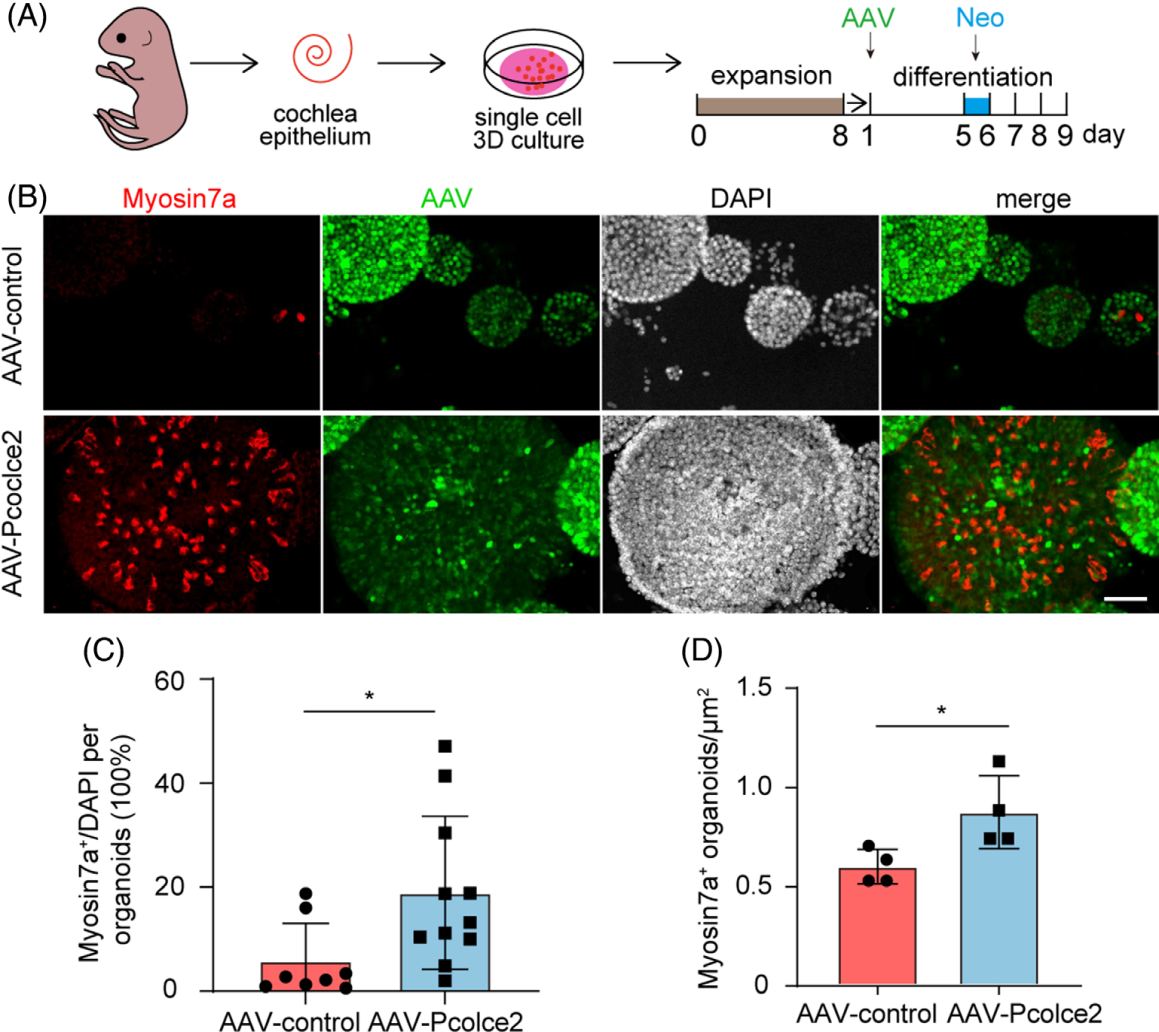
Procollagen C‐endopeptidase enhancer 2 (*Pcolce2*) overexpression promotes hair cell production in damaged three‐dimension (3D) culture. (A) Flow chart of the differentiation culture experiments after organoid injury. Inner ear stem cells were proliferated for 8 days, differentiated and cultured, and then transfected with AAV‐control and AAV‐*Pcolce2*. After 5 days of differentiation, neomycin injury (0.5 mM) was performed for 1 day, and the culture was continued for 3 days. (B) Myosin7a (red) immunofluorescence staining images of the AAV‐transfected organoids. Scale bar: 100 μm. (C) The percentage of Myosin7a^+^ cells/DAPI of each organoid in (B). (D) Myosin7a^+^ cells of organoids in each well in (B). *p < 0.05.

Previous studies indicated that SCs can differentiate into HCs in models of HC damage.^40^ Aminoglycosides such as neomycin can cause damage to inner ear HCs,^41^ thus we investigated the effect of *Pcolce2* overexpression in the neomycin‐damaged cochlear organoids (Figure 4A). Myosin7a staining showed that the ratio of Myosin7a^+^/DAPI cells and the number of Myosin7a^+^ organoids significantly number increased after *Pcolce2* overexpression (Figure 4B–D). These results suggest that overexpression of *Pcolce2* can promote organoid differentiation in organoid culture after neomycin injury.

We then performed an explant culture to determine whether overexpression of *Pcolce2* can also promote HC regeneration after injury (Figure S3A). We stained the cultured explants for EdU and Myosin7a (Figure S3B), but no EdU^+^/Myosin7a^+^ cells were detected in either experimental or control groups. Also, no obvious increased number of HCs was seen in *Pcolce2* overexpressing cochlear explants (Figure S3C). We also conducted a neomycin injury experiment after overexpression of *Pcolce2* in vivo (Figure S4A) and found that compared to the control group, overexpression of *Pcolce2* did not improve hearing (Figure S4B). According to imaging and statistical analysis, the numbers of inner HCs, outer HCs and total HCs were not significantly different in the cochlea (Figure S4C–F). These results were inconsistent with the stem cell injury results (Figure 4), and this might be due to the stem cell culture medium containing LY (LY411575, an inhibitor of Notch signalling) and C (CHIR99021, an agonist of Wnt signalling).

### 3.5 *Pcolce2* combined with LY and C promotes HC differentiation by activating Wnt pathways after injury

**FIGURE 5:**
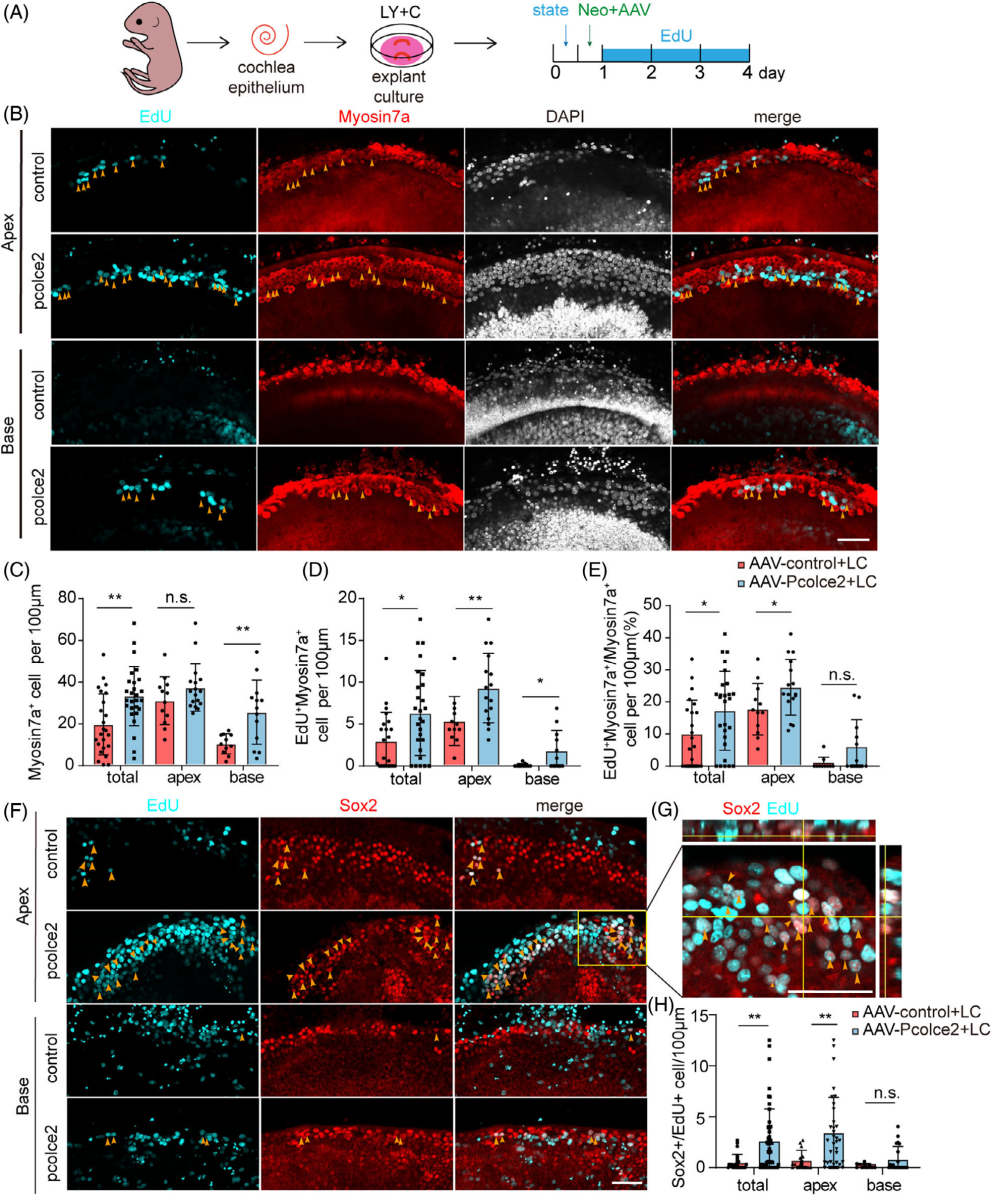
Procollagen C‐endopeptidase enhancer 2 (*Pcolce2*) overexpression promotes the production of hair cell (HCs) in damaged neonatal mammalian cochleae in vitro. (A) Experimental plan of the injured cochlear explant culture in vitro. The cochlear basement membrane was stable for 12 h after dissection, AAV and neomycin were added at the same time for 12 h, and the membranes were cultured for 3 additional days in the presence of EdU. AAV dose: 2 × 10^10^ GCs/cochlea. LY (5 μM, LY411575, Notch inhibitor), and C (3 μM, CHIR99021, Wnt agonist). (B) Immunostaining images of EdU (cyan) and Myosin7a (red) signals in AAV‐control and AAV‐*Pcolce2*‐transfected cochlear explants. Yellow arrows indicate the EdU^+^/Myosin7a^+^ HCs. Scale bar: 50 μm. (C) Myosin7a^+^ cells per 100 μm in (B). (D) EdU^+^/Myosin7a^+^ HCs per 100 μm in (B). (E) The percentage of double‐positive EdU^+^/Myosin7a^+^ HCs per 100 μm in (B). (F) The Sox2 (Red) and EdU (Cyan) immunostaining images of cultured explant in (A). Arrows indicate some of the Sox2^+^/EdU^+^ SCs. Scale bar: 50 μm. (G) Magnified images and their orthogonal views of the yellow boxed region in (F). Scale bar: 50 μm. (H) EdU^+^/Myosin7a^+^ cells per 100 μm in (F). *p < 0.05, **p < 0.01, n.s. refers to no significance. C, CHIR99021; L, LY411575; LC, LY+C.

**FIGURE 6:**
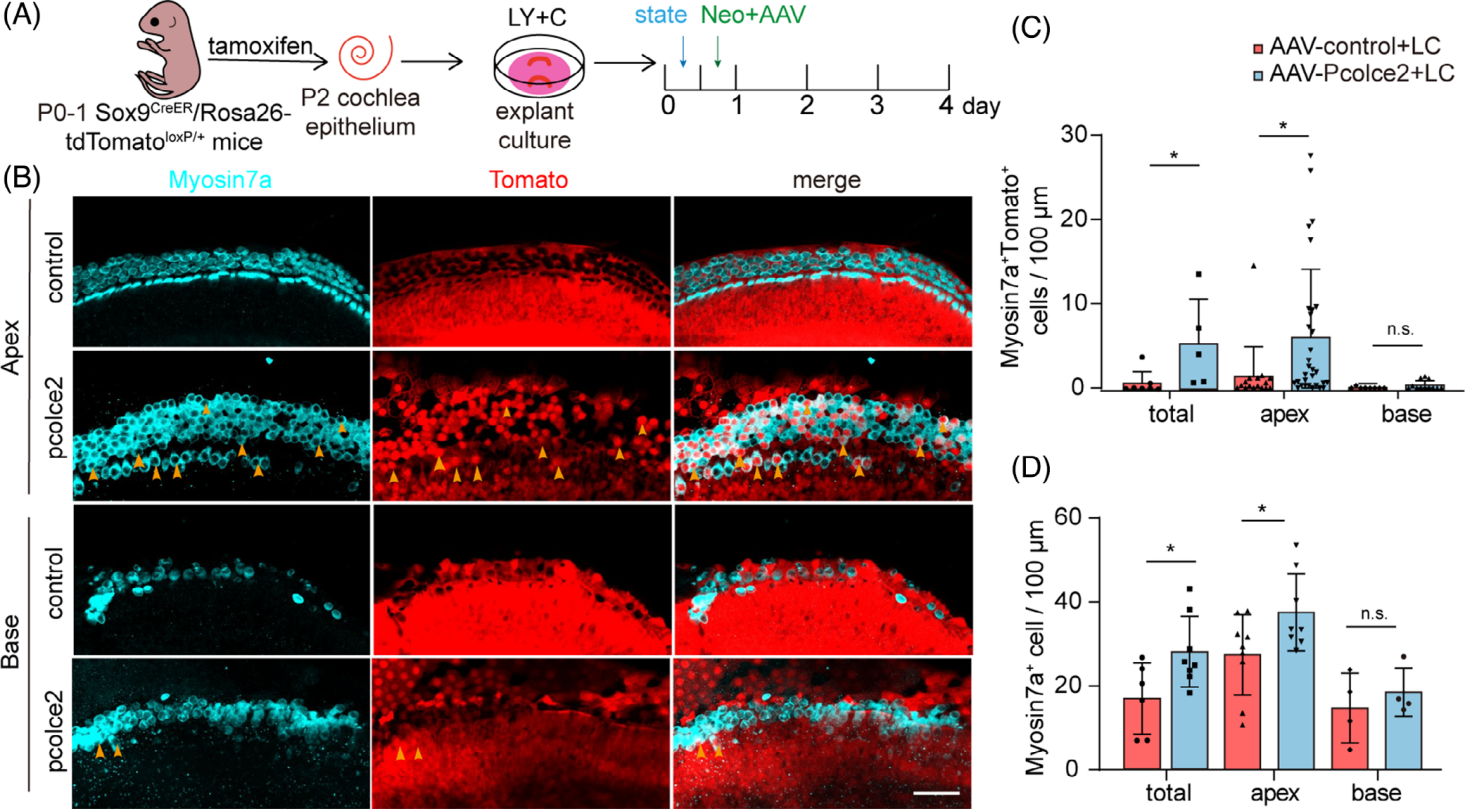
Procollagen C‐endopeptidase enhancer 2 (*Pcolce2*) overexpression promotes the production of hair cell (HCs) from Sox9^+^ supporting cells in damaged neonatal mammalian cochleae. (A) Experimental plan. Activation of tomato expression by tamoxifen injection in mice at age p0‐p1 and dissociation of the inner ear basilar membrane the next day, AAV and neomycin were added for 12 h, and then cultured for 3 additional days. AAV dose: 2 × 10^10^ GCs/cochlea. (B) Immunostaining images of Myosin7a (cyan) and tomato signals in AAV‐control and AAV‐*Pcolce2*‐transfected cochlear explants. White arrows indicate the Myosin7a^+^/Tomato^+^ HCs. Scale bar: 50 μm. (C) Myosin7a^+^/Tomato^+^ HCs per 100 μm in (B). (D) Myosin7a^+^ cells per 100 μm in (B). *p < 0.05, n.s. refers to no significance.

Simultaneously regulating the Wnt and Notch signalling pathways can improve HC regeneration.^19^ Therefore, we also added LY and C to the explant culture medium to further examine the effect of *Pcolce2* on HC regeneration in the cochlear explant injury model (Figure 5A). The number of surviving HCs in the *Pcolce2* overexpression group was greater than that in the control (Figure 5B,C). Moreover, proliferating HCs were marked by EdU and Myosin7a (Figure 5B), and the number of proliferating HCs and the proportion of proliferating HCs were increased in the *Pcolce2* overexpressing cochlea compared to the control group (Figure 5D,E). We examined the proliferating cells with EdU and found that overexpression of pcolce2 significantly promoted the proliferation of the SCs (Figure 5F–H). This suggests that the proliferating HCs may have originated from the SCs. Next, cochlear tissue from Sox9^CreER^/Rosa26‐tdTomato^loxp/loxp^ mice was used for explant damage culture in vitro (Figure 6A,B). Regenerated HCs derived from Sox9^+^ SCs, also known as Myosin7a^+^Tomato^+^ cells, and the number of regenerated HCs were tracked and counted. We found that *Pcolce2* overexpression significantly increased the number of tracked Myosin7a^+^Tomato^+^ cells and improved the survival of HCs (Figure 6C,D). The above results indicated that *Pcolce2* promoted HC regeneration after injury in the presence of Wnt signalling agonists and Notch signalling inhibitors.

**FIGURE 7:**
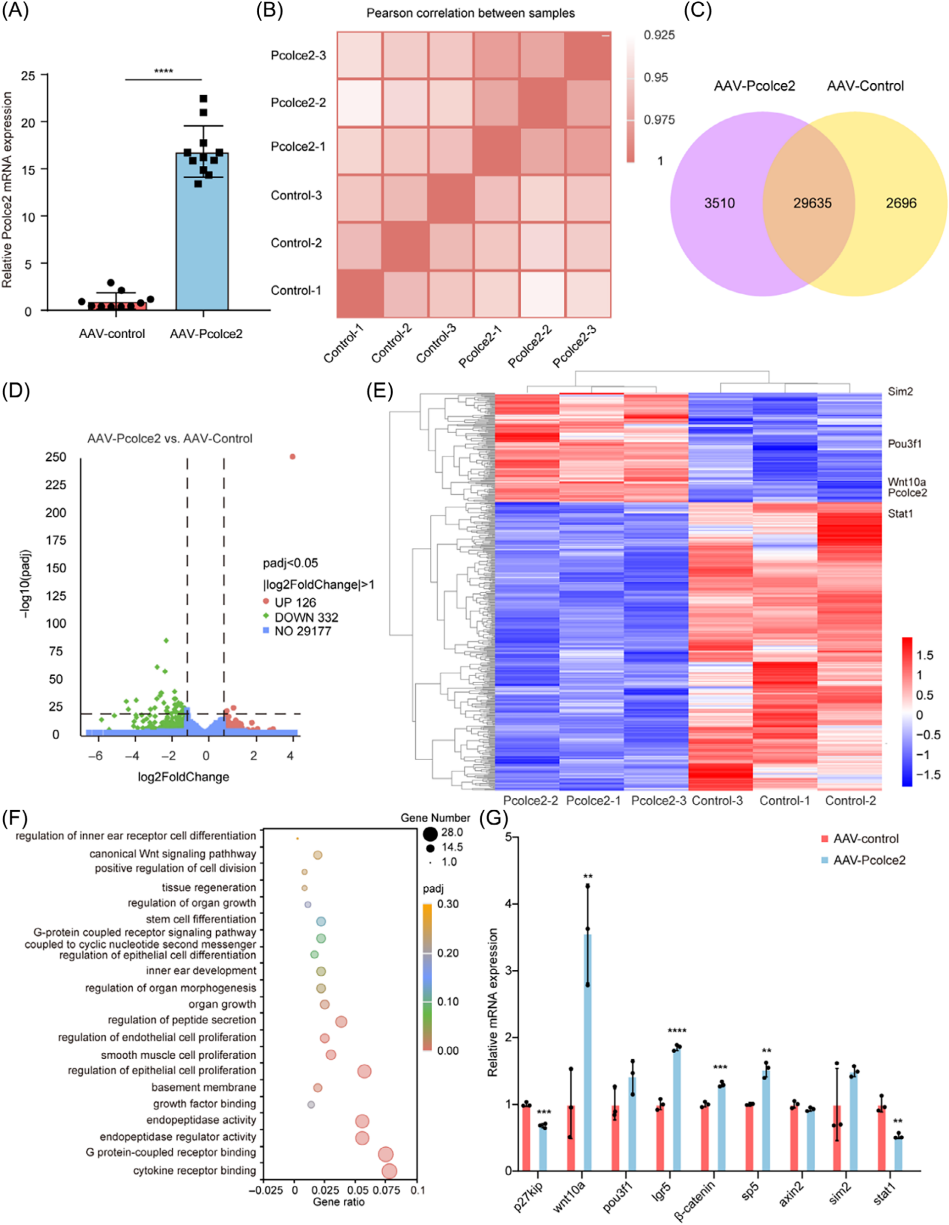
RNA‐seq analysis after procollagen C‐endopeptidase enhancer 2 (*Pcolce2*) overexpression in damaged neonatal mammalian cochleae in vitro. (A) The quantitative real‐time PCR (qPCR) results of *Pcolce2* overexpression in the AAV‐*Pcolce2‐*transfected neomycin‐damaged cochlear explants. (B) Pearson correlation between the AAV‐control and the AAV‐*Pcolce2*. (C) Venn chart results of *Pcolce2* overexpression and controls. (D) Differential gene expression volcano plot for *Pcolce2* overexpression and controls. (E) The result of differential gene clustering analysis between AAV‐control and AAV‐*Pcolce2*. (F) Gene ontology analysis of differentially expressed genes related to inner ear development and related signalling pathways between AAV‐control and *Pcolce2*. (G) The qPCR results of downstream genes derived from sequencing results in the AAV‐control and *Pcolce2* groups. **p < 0.01, ***p < 0.001 and ****p < 0.0001.

To explore the mechanism behind these observations, we further performed RNA sequencing analysis on the cochlear explant samples in Figure 5. As measured by qPCR, *Pcolce2* was found to be 17‐fold overexpressed at the RNA level (Figure 7A), and the Pierce coefficient between replicate samples showed little difference (Figure 7B). There was a total of 29,635 co‐expressed genes in the *Pcolce2* overexpression and the control group (Figure 7C). The volcano plot in Figure 7D shows the differentially expressed genes, and of these, there were 126 up‐regulated genes, such as *Sim2*, *Pou3f1*, *Wnt10a* and the target gene *Pcolce2*, and 332 down‐regulated genes, such as *Stat1* (Figure 7E). Through Gene Ontology analysis, we found that the differentially expressed genes were related to inner ear receptor cell differentiation and the classic Wnt signalling pathway (Figure 7F). We performed qPCR to verify the related genes, the cell cycle repressor P27^kip^ was down‐regulated, and the Wnt downstream genes *Wnt10a*, *Lgr5*, *β‐catenin* and *Sp5* were up‐regulated, demonstrating that *Pcolce2* overexpressed activated the Wnt pathways in the damaged cochlear explant (Figure 7G). These results suggest that the overexpression of *Pcolce2* in the presence of LY and C can promote HC regeneration after cochlear basilar membrane injury.

## 4 DISCUSSION

An ideal therapeutic method for treating SNLH is to regenerate HCs from stem cells or cochlear progenitor cells to restore the structure and function of the cochlea. Therefore, HC regeneration is the focus of hearing recovery research.^42^, ^43^ Studies have shown that in the mouse cochlea, SCs have the potential to differentiate into HCs, and this occurs either by SCs first proliferating and then differentiating into HCs^12^ or by the direct trans‐differentiating SCs into HCs.^44^ Many studies have focused on regulating the differentiation of SCs into HCs, including *Atoh1* factor‐induced ectopic expression of HCs, etc.^45^, ^46^, ^47^, ^48^ In this study, we revealed a potential role for *Pcolce2* in HC regeneration.

Much research has demonstrated that multiple signalling pathways are involved in the development of the inner ear.^7^, ^16^, ^49^ Wnt activation can reprogramme SCs for expansion and differentiation into HCs.^50^ *Axin2* and *Lgr5* are two downstream target genes of Wnt.^51^ *Lgr5* expression is characteristic of inner ear progenitor cells,^22^ but *Lgr5* expression in the cochlea decreases with age in mice, and the cells progressively lose stem cell properties.^8^ Consistent with this, we discovered that *Axin2* and *Lgr5* mRNA expression was increased in the *Pcolce2*‐overexpressing mouse cochlea. In addition, *Wnt10a*, one of the 19 known Wnt ligands, has been shown in many studies to participate in cancer cell proliferation and invasion through activating Wnt/β‐catenin signalling.^52^, ^53^, ^54^ Our qPCR results also showed Wnt/β‐catenin downstream gene, including *Lgr5*, *Wnt10a*, *beta‐catenin* and *Sp5*, and these all increased after overexpression of *Pcolce2* in neomycin‐damaged cochlear explants. *P27* ^*Kip1*^ is a cyclin‐dependent kinase inhibitor that coordinates cell proliferation and morphogenesis.^55^ Downregulation of *P27* ^*Kip1*^ has been shown to contribute to the organ of Corti's mitosis, thus promoting the growth regulation of cell proliferation.^56^ In the present study, overexpression of *Pcolce2* down‐regulated the expression of *P27* ^*Kip1*^ and promoted SC proliferation in the neomycin‐damaged model. Thus, we found that *Pcolce2* overexpression can enhance cochlear progenitors' proliferation by activating multiple signalling pathways and regulating HC regeneration in the inner ear through the Wnt/β‐catenin signalling pathway.

Aminoglycosides, such as neomycin, are often used as clinically beneficial drugs, but they can have serious side effects and are regarded as ototoxic drugs,^57^, ^58^, ^59^ mainly causing loss of vestibular function and permanent hearing impairment.^60^ Studies have shown that biological processes of injury and repair exist simultaneously in damaged cochleae.^13^ In this study, we observed a similar phenomenon in the organoid cultures in vitro, and *Pcolce2* induced an increase in nascent HCs after injury.

Similarly, HC regeneration is a process involving the coordinated regulation of multiple signalling pathways and small molecule drugs. LY is an inhibitor of Notch signalling, and C is an agonist of Wnt signalling, and the combination of LY and C has been studied to promote the expansion and differentiation of cochlear organoids in vitro.^61^ Similarly, in our study, the combination of *Pcolce2*, LY and C could promote HC regeneration in the neomycin‐damaged cochlear explants. However, in our subsequent neomycin injury experiments in mice, no significant recovery effect was observed in terms of the number of HCs or hearing function (Figure S5). This suggests that the effect of *Pcolce2* may need to be combined with multiple genes and signalling pathways to result in functional HC regeneration.

In summary, in this study, we provide evidence that in the neonatal mouse cochlea, AAV‐ie mediated *Pcolce2* promotes SC reprogramming into HCs by activating Wnt/β‐catenin signalling. AAV‐mediated gene therapy, which combines *Pcolce2* with multiple signalling pathways, is expected to be a new therapeutic strategy for deafness in the clinic.

## AUTHOR CONTRIBUTIONS

BT, JS, RC, and YS conceived and designed the experiments. CX, LZ, YZ, and HD performed most of the experiments. CX contributed to the data analysis. LP performed data processing for single‐cell sequencing. XG, NL, QS, ZZ, YL, and XQ helped with the experiments and the data analysis. CX, LZ, JQ, and FT discussed the data analysis, interpretation, and presentation and wrote the manuscript with contributions from all authors.

## FUNDING INFORMATION

This work was supported by the National Key Research and Development Program of China (2021YFA1101300 [Renjie Chai], 2021YFA1101800 [Renjie Chai], 2020YFA0113600 [Jieyu Qi], and 2020YFA0112503 [Renjie Chai]), the CAMS Innovation Fund for Medical Sciences (2019‐12M‐5‐032 [Yi Shi]), the STI2030‐Major Projects (2022ZD0205400), the National Natural Science Foundation of China (82271120 [Yi Shi], 82201234 [Yi Shi], 82121003 [Yi Shi],82000984 [Jieyu Qi], 82030029 [Renjie Chai], 92149304 [Renjie Chai], 82371162 [Renjie Chai], 82371161 [Renjie Chai] and 82371156 [Renjie Chai]), the Science and Technology Department of Sichuan Province (2022ZYD0066 [Yi Shi], 2022YFS0606 [Yi Shi] and 2021YFS0371 [Renjie Chai]), the Natural Science Foundation of Anhui Province(2208085MH231 [Jiaqiang Sun]), the Shenzhen Science and Technology Program (JCYJ20210324125608022 [Renjie Chai] and JCYJ20190814093401920 [Renjie Chai]), the Open Research Fund of State Key Laboratory of Genetic Engineering, Fudan University (SKLGE‐2104 [Renjie Chai]), the 2022 Open Project Fund of Guangdong Academy of Medical Sciences to P.N.W. (YKY‐KF202201 [Renjie Chai]) and the Fundamental Research Funds for the Central Universities.

## CONFLICT OF INTEREST STATEMENT

Jieyu Qi and Fangzhi Tan have filed a patent on the use of AAV‐ie for gene therapy in the inner ear. The other authors have no other conflict of interest to declare.

## Supporting information


**Figure S1.** The expression of *Pcolce2* in the mouse cochlea.
**Figure S2.** Overexpression of *Pcocle2* had no obvious effect on HC regeneration in wild‐type mice.
**Figure S3.** Overexpression of *Pcolce2* failed to promote HC regeneration in the explant culture injury model in vitro.
**Figure S4.** *Pcolce2* failed to promote HC regeneration in an injury model in vivo.
**Figure S5.** *Pcolce2* combined with Wnt agonist and Notch inhibitor failed to promote HC regeneration in the neomycin‐damaged model in vivo.

**Table S1.** Primers used for qPCR.

## Data Availability

All data associated with this study are present in the paper or the supplementary materials.
