# Supplementary material for: Pcolce2 overexpression promotes supporting cell reprogramming in the neonatal mouse cochlea
Source: Cell Prolif. 2024 Mar 25;57(8):e13633. doi: 10.1111/cpr.13633 (PMC11294419; doi:10.1111/cpr.13633)
Supplement: Supplementary file 1 — Figure S1. The expression of Pcolce2 in the mouse cochlea. Figure S2. Overexpression of Pcocle2 had no obvious effect on HC regeneration in wild‐type mice. Figure S3. Overexpression of Pcolce2 failed to promote HC regeneration in the explant culture injury model in vitro. Figure S4. Pcolce2 failed to promote HC regeneration in an injury model in vivo. Figure S5. Pcolce2 combined with Wnt agonist and Notch inhibitor failed to promote HC regeneration in the neomycin‐damaged model in vivo. [file CPR-57-e13633-s002.docx]

**Supplementary Materials for**

***Pcolce2* overexpression promotes supporting cell reprogramming in the neonatal mouse cochlea**

Changling Xu^1,2,3,#^, Liyan Zhang^4,#^, Yinyi Zhou^4,#^, Haoliang Du^5,#^, Jieyu Qi^4,#^, Fangzhi Tan^4,#^, Li Peng^6^, Xingliang Gu^4^, Nianci Li^4^, Qiuhan Sun^4^, Ziyu Zhang ^4^,Yicheng Lu^4^, Xiaoyun Qian^7^, Busheng Tong^8*^, Jiaqiang Sun^9*^, Renjie Chai^4,10,11,12,13*^, Yi Shi^1,2,3*^

^*^Corresponding author: tongbusheng@ahmu.edu.cn, sunjq0605@126.com, renjiec@seu.edu.cn, and shiyi1614@126.com.

The PDF file includes:

Fig. S1. The expression of *Pcolce2* in the mouse cochlea.

Fig. S2. Overexpression of *Pcocle2* had no obvious effect on HC regeneration in wild-type mice.

Fig. S3. Overexpression of *Pcolce2* failed to promote HC regeneration in the explant culture injury model *in vitro*.

Fig. S4: *Pcolce2* failed to promote HC regeneration in an injury model *in vivo*.

Fig. S5: *Pcolce2* combined with Wnt agonist and Notch inhibitor failed to promote HC regeneration in the neomycin-damaged model *in vivo.*


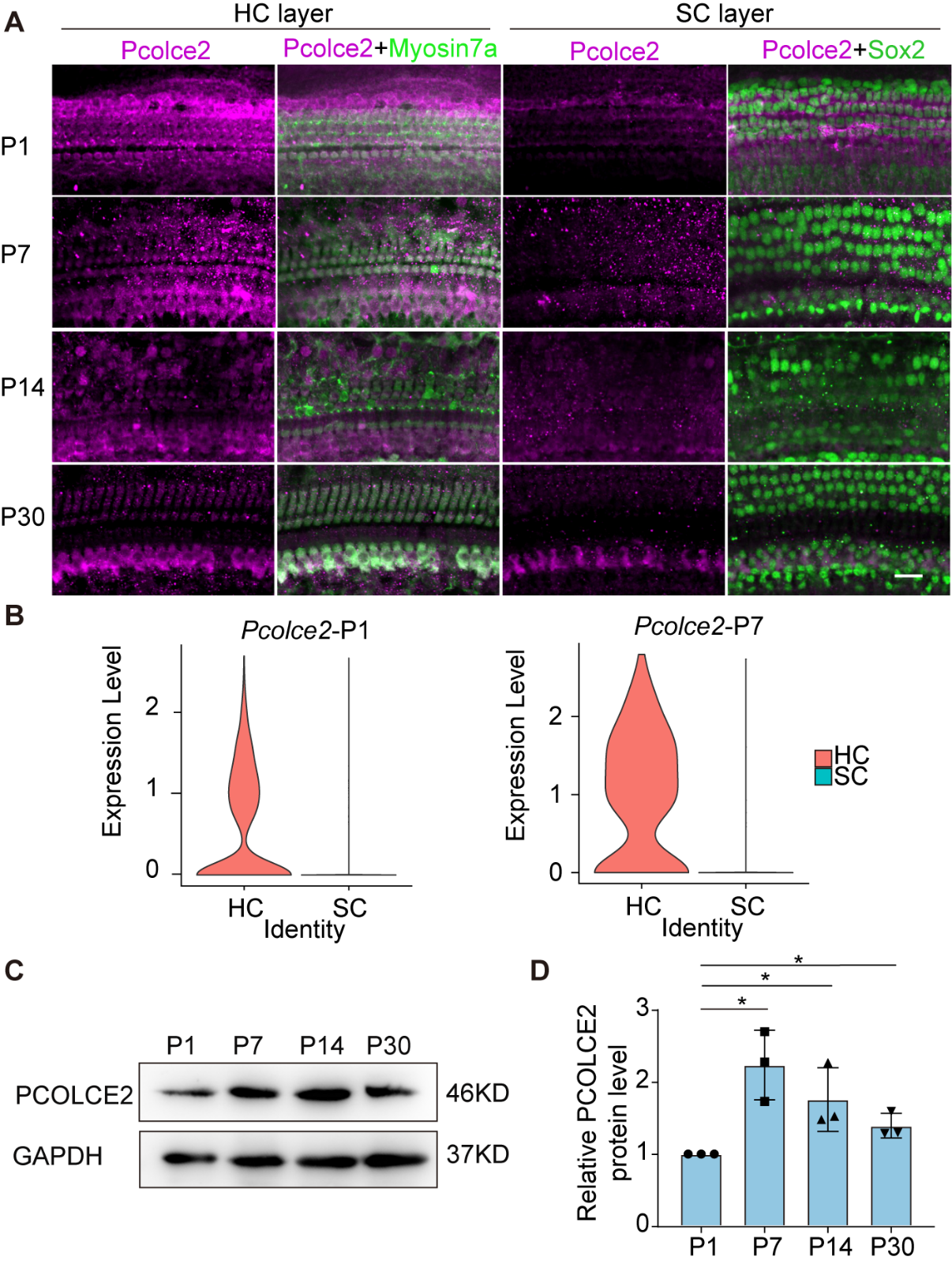


**Figure S1. The PCOLCE2 protein level and expression of *Pcolce2* in the mouse cochlea.**

**(A)** The immunostaining images for PCOLCE2 (magenta) in mice cochlear epithelia at ages P1, 7, 14, and 30. Myosin7a (shown in green on the left) marks HCs, and SOX2 (green on the right) marks SCs. Scale bar: 50 μm.

**(B)** Violin plots showing the expression levels of *Pcolce2* in SC and HC cells at two time points.

**(C)** Western blot for PCOLCE2 in the mice cochlea from age P1, 7, 14, and 30.

**(D)** The protein level quantified of PCOLCE2 in the mice cochlea from age P1, 7, 14, and 30. **p* < 0.05.


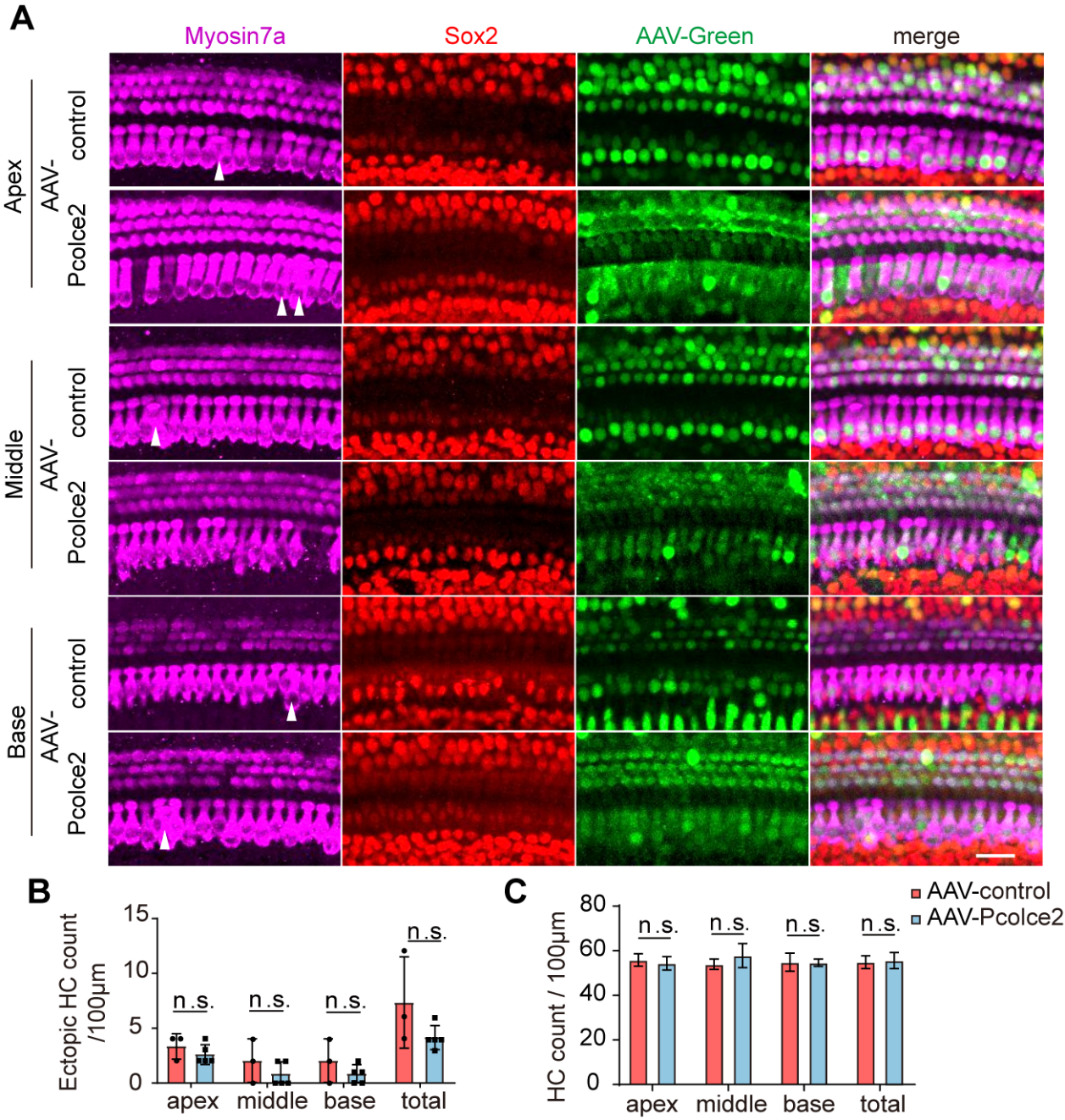


**Figure S2. Overexpression of *Pcocle2* had no obvious effect on HC regeneration in wild-type mice.**

**(A)** Immunostaining images of Myosin7a (magenta) in three turns (apical, middle, and basal) of mice cochlea transduced by AAV-control and *Pcolce2*. Sox2 (red) marks SCs. AAV-transfected cells are in green. White arrows indicate ectopic HCs. AAV dose: 9 × 10^10^ GCs/cochlea. Scale bar: 50 μm.

**(B)** Ectopic HCs in (A).

**(C)** The number of HCs per 100 μm in (A). n.s. refers to no significance.


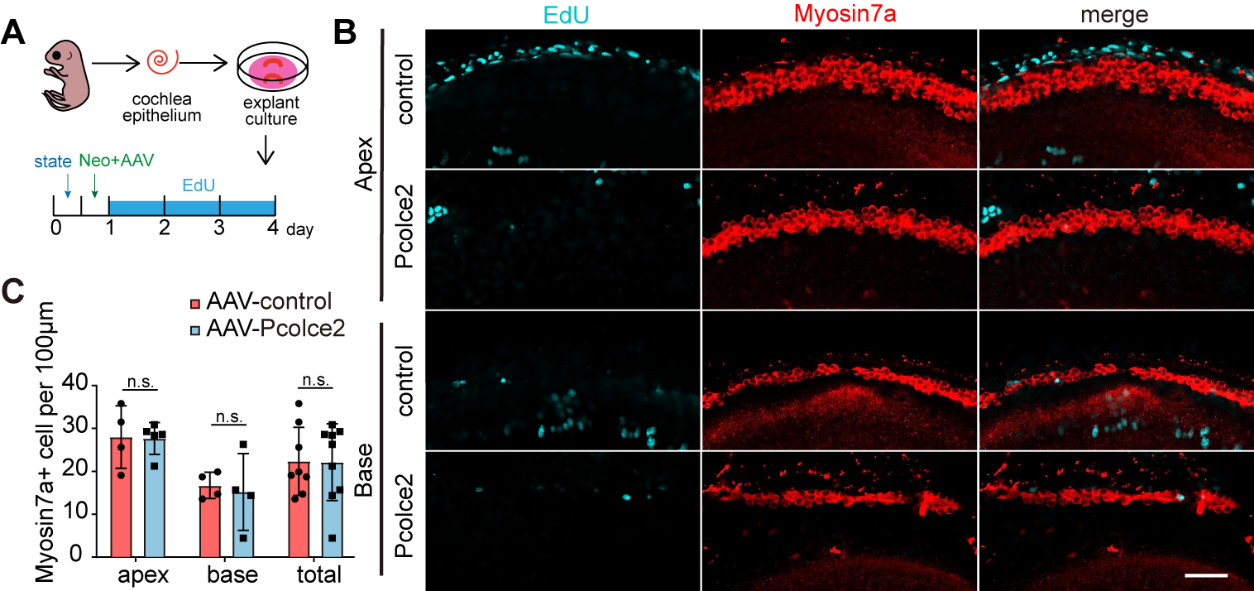


**Figure S3. Overexpression of *Pcolce2* failed to promote HC regeneration in the explant culture injury model *in vitro*.**

**(A)** Flow chart of the cochlear explant culture after neomycin injury. The cochlear basilar membrane was obtained from P2-P3 mice, cultured *in vitro*, transfected with AAV-*Pcolce2*, damaged with neomycin, and cultured with EdU for 3 days. AAV dose: 2 × 10^10^ GCs/cochlear explant.

**(B)** Immunofluorescence staining of EdU (cyan) and Myosin7a (red) after injury in cultured explants overexpressing control and *Pcolce2*. Scale bar: 100 μm.

**(C)** Myosin7a^+^ cells per 100 μm. n.s. refers to no significance.


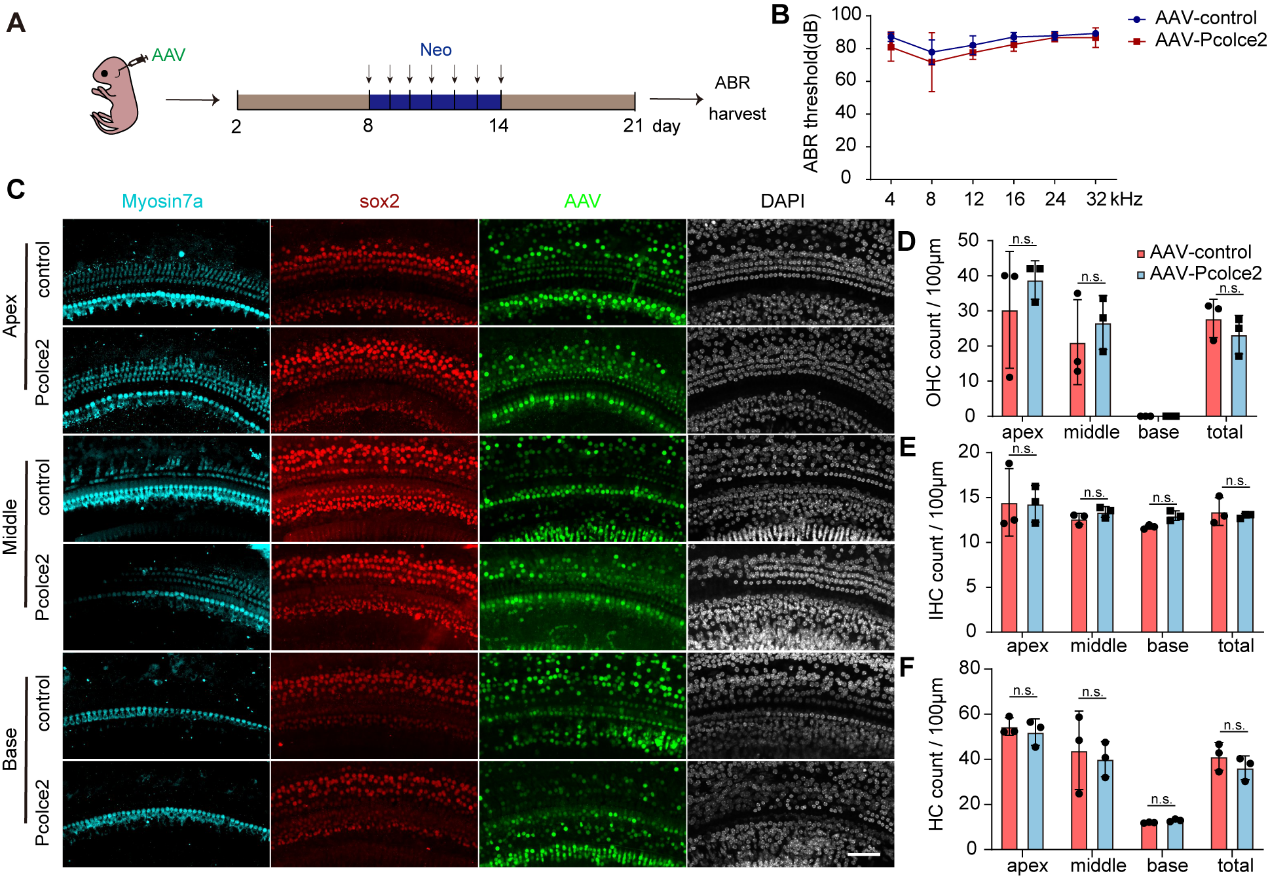


**Figure S4. *Pcolce2* failed to promote HC regeneration in an injury model *in vivo*.**

**(A)** The experimental plan of the *in vivo* cochlear HC damage model*.* The mice were injected with the virus at P1-P2, neomycin was injected at P8-P14, and ABR results and samples were collected at P21. AAV dose: 4.5 × 10^10^ GCs/cochlea.

**(B)** ABR measurement results of P21 AAV injected mice.

**(C)** Representative immunostaining of Myosin7a (cyan) and Sox2 (red) in the cochlea after harvest (AAV in green). Scale bar: 50 μm.

**(D-F)** The number of outer HCs (D), inner HCs (E), and total HCs (F) per 100 μm in the cochleae from (B). n.s. refers to no significance.

**
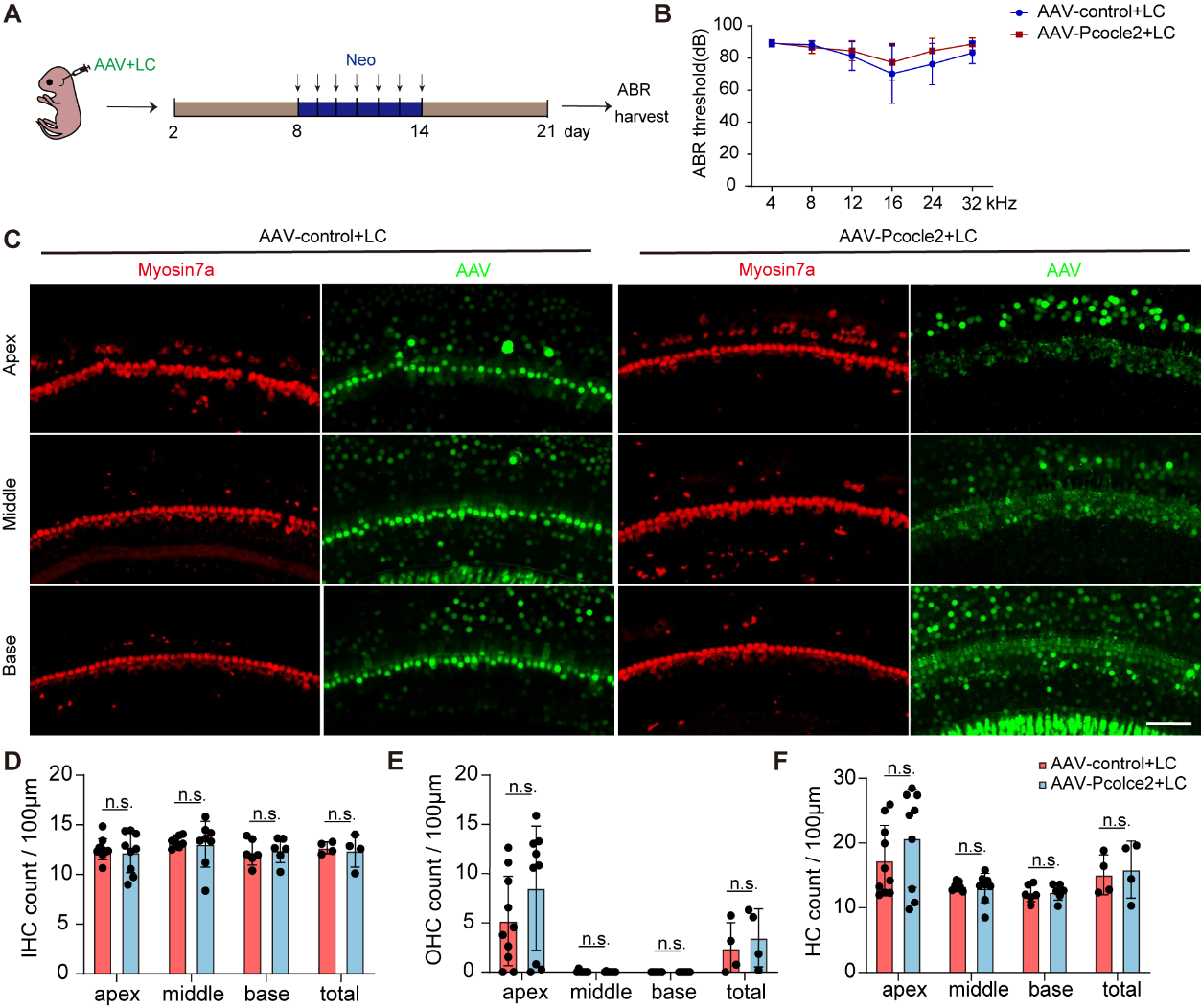
**

**Figure S5. *Pcolce2* combined with Wnt agonist and Notch inhibitor failed to promote HC regeneration in the neomycin-damaged model *in vivo*.**

**(A)** The experimental plan of cochlear HC injury *in vivo*. L: LY411575 (Notch inhibitor), C: CHIR99021(Wnt agonist).

**(B)** ABR measurement results in P21 AAV injected mice.

**(C)** Representative immunostaining of Myosin7a (cyan) in the cochlea after harvest. Scale bar: 100 μm.

**(D-F)** The count of inner HCs (D), outer HCs (E), and total HCs (F) per 100 μm in (B). n.s. refers to no significance.
