# Supplementary material for: Pcolce2 overexpression promotes supporting cell reprogramming in the neonatal mouse cochlea
Source: Cell Prolif. 2024 Mar 25;57(8):e13633. doi: 10.1111/cpr.13633 (PMC11294419; doi:10.1111/cpr.13633)
Supplement: Supplementary file 2 — Table S1. Primers used for qPCR. [file CPR-57-e13633-s001.docx]

**Supplementary Materials**

**Table S1. Primers used for qPCR.**

| Gene name | Forward primer (5′-3′) | Reverse Primer (5′-3′) |
| --- | --- | --- |
| tdTomato- wild-type | AAGGGAGCTGCAGTGGAGTA | CCGAAAATCTGTGGGAAGTC |
| tdTomato- mutant | GGCATTAAAGCAGCGTATCC | CTGTTCCTGTACGGCATGG |
| Sox9- mutant | GCGGTCTGGCAGTAAAAACTATC | GTGAAACAGCATTGCTGTCACTT |
| Sox9-  wild-type | CTAGGCCACAGAATTGAAAGATCT | GTAGGTGGAAATTCTAGCATCATCC |
| ITR | GGAACCCCTAGTGATGGAGTT | CGGCCTCAGTGAGCGA |
| Gapdh | AGGTCGGTGTGAACGGATTTG | TGTAGACCATGTAGTTGAGGTCA |
| Pcolce2 | TGTGGCGGCATTCTTACCG | CCCTCAGGAACTGTGATTTTCCA |
| Axin2 | TGACTCTCCTTCCAGATCCCA | TGCCCACACTAGGCTGACA |
| Lgr5 | CCTACTCGAAGACTTACCCAGT | GCATTGGGGTGAATGATAGCA |
| β-catenin | ATGCGCTCCCCTCAGATGGTGTC | TCGCGGTGGTGAGAAAGGTTGTGC |
| Hes1 | ACGACACCGGACAAACCA | ATGCCGGGAGCTATCTTTCT |
| Jag1 | TGTGCAAACATCACTTTCACCTTT | GCAAATGTGTTCGGTGGTAAGAC |
| Notch1 | GGAGGACCTCATCAACTCACA | CGTTCTTCAGGAGCACAACA |
| Hey1 | CACTGCAGGAGGGAAAGGTTAT | CCCCAAACTCCGATAGTCCAT |
| Gli3 | TTTTCCCTGCCTTCCATCCT | ATTACGGTGTGGGGAGATCC |
| Ptch1 | ATGGCCGCATTGATCCCTAT | TCTTCTGTCCTCACGTCTGT |
| Gli2 | ACTCTCACCTCCATCAGCAC | CTCAGCCTCAGTCTTGACCT |
| Hhip | TCCCGAGAAAGCAAGTCAGA | GCCCACTCATGACCTCCTG |
| Gli1 | TCTCCGACCCCTCCACAG | AGAGATCCTTCAGTGCAGCT |
| P27^kip^ | CGGTGCCTTTAATTGGGTCT | AGCAGGTCGCTTCCTCATC |
| Wnt10a | GCTCAACGCCAACACAGTG | CGAAAACCTCGGCTGAAGATG |
| Pou3f1 | TCGAGGTGGGTGTCAAAGG | GGCGCATAAACGTCGTCCA |
| Sp5 | CGGACCTGGGCAAGCACT | GGGTGGAAAAGTCTGGAGGG |
| Notch3 | TGCCAGAGTTCAGTGGTGG | CACAGGCAAATCGGCCATC |
| Hes5 | TGCTCAGTCCCAAGGAGAAA | AGCTTGGAGTTGGGCTGGT |
| Sim2 | CACAGCAATACAGCTCCTTCC | CGGCCCATATAAAAGGTCGCT |
| Stat1 | TCACAGTGGTTCGAGCTTCAG | GCAAACGAGACATCATAGGCA |
